# External validation of the ROSC after cardiac arrest (RACA) score in a physician staffed emergency medical service system

**DOI:** 10.1186/s13049-017-0380-2

**Published:** 2017-03-29

**Authors:** Petteri Kupari, Markus Skrifvars, Markku Kuisma

**Affiliations:** 1 Emergency Medicine, Section of EMS, University of Helsinki and Department of Emergency Medicine and Services, Helsinki University Hospital, P.O. Box 112, FIN-00099 Helsingin kaupunki, Finland; 2 Division of Intensive care, Department of Anaesthesiology, Intensive Care and Pain Medicine, Helsinki University Hospital and Helsinki University, Meilahden sairaala, Haartmaninkatu 4, FIN-00029 HUS Helsinki, Finland; 3 Australian and New Zealand Intensive Care Research Centre, School of Public Health and Preventive Medicine, Monash University, Melbourne, VIC Australia

**Keywords:** Cardiac arrest, Resuscitation, RACA score, Emergency medical service

## Abstract

**Background:**

The return of spontaneous circulation (ROSC) after cardiac arrest (RACA) score may have implications as a quality indicator for the emergency medical services (EMS) system. We aimed to validate this score externally in a physician staffed urban EMS system.

**Methods:**

We conducted a retrospective cohort study. Data on resuscitation attempts from the Helsinki EMS cardiac arrest registry from 1.1.2008 to 31.12.2010 were collected and analyzed. For each attempted resuscitation the RACA score variables were collected and the score calculated. The endpoint was ROSC defined as palpable pulse over 30 s. Calibration was assessed by comparing predicted and observed ROSC rates in the whole sample, separately for shockable and non-shockable rhythm, and separately for resuscitations lead by a specialist, registrar or medical supervisor (i.e., senior paramedic). Data are presented as medians and interquartile ranges. Statistical testing included chi-square test, the Mann-Whitney *U* test, Hosmer-Lemeshow goodness of fit test and calculation of 95% confidence intervals (CI) for proportions.

**Results:**

A total of 680 patients were included of whom 340 attained ROSC. The RACA score was higher in patients with ROSC (0.62 [0.46–0.69] than in those without (0.46 [0.36–0.57]) (*p* < 0.001). Observed against predicted ROSC indicated reasonable calibration overall (*p* = 0.30), with better calibration in patients with a shockable initial rhythm (*p* = 0.75) than in patients with a non-shockable rhythm (*p* = 0.04). There was no statistical difference between observed and predicted ROSC rates in resuscitations attended by a specialist (50% vs 53%, 95% CI 45–55) or registrar (55% vs 53%, 95% CI 48–62), but rates were lower than predicted in resuscitations lead by a medical supervisor (36% vs 49%, 95% CI 25–47).

**Discussion:**

Developing a practical severity-of-illness scoring system for out-of-hospital cardiac arrest patients would allow patient heterogeneity adjustment and measurement of quality of care in analogy to commoly used severity-of-illness- scores developed for the similar purposes for the general intensive care unit population. However, transferring RACA score to another country with different population and EMS system might affect the performance and generalizability of the score.

**Conclusions:**

This study found a good overall calibration and moderate discrimination of the RACA score in a physician staffed urban EMS system which suggests external validity of the score. Calibration was suboptimal in patients with a non-shockable rhythm which may due to a local do-not-attempt-resuscitation policy. The lower than expected overall ROSC rate in resuscitations attended by medical supervisors requires further study.

## Background

The incidence of out-of hospital cardiac arrest (OHCA) in Europe is estimated to be 37-55/100,000 inhabitants per year [1, 2]. Because of the major burden that OHCA patients cause to EMS systems and hospitals worldwide quality assurance is paramount [3]. This has resulted in attempts to develop means for predicting survival and comparing pre-hospital care of OHCA patients [4–11]. Outcome of OHCA vary: in high-quality EMS systems spontaneous circulation may be achieved in up to 53% of patients at least until hospital admission and discharge rates are reported to be between 14 and 20% while much lower rates are reported from other EMS systems [1, 3, 12, 13]. The comparability of different cohorts has been questioned and direct outcome comparisons may also be affected by alternating definitions of inclusion and exclusion criteria [4]. There seems to be a considerable need for practical scoring system allowing comparison between these different EMS systems and patient cohorts and thus, serving as a quality indicator.

In 2011 Gräsner and colleagues developed and internally validated a score to predict occurrence of return of spontaneous circulation (ROSC) after out-of hospital cardiac arrest, the so called return of spontaneous circulation after cardiac arrest (RACA) score [4]. The return of spontaneous circulation after cardiac arrest core was developed with data from the German Resuscitation Registry and incorporates multiple pre-resuscitation variables found to have a significant positive or negative impact on the probability of ROSC. Importantly, the RACA score is based on variables available on EMS arrival at the scene; e.g., patient age, gender, aetiology, place and EMS delay. However, RACA score is not designed to be calculated at the scene, influence medical management or to be used as a prognostic tool, in order to determine the success or failure of resuscitation of individual patient.

Since the original study was performed with data from the German Resuscitation Registry, its applicability in other countries with different EMS systems and populations is currently unknown. The aim of this study was to validate externally the RACA score in a physician staffed urban EMS system. We hypothesized that the RACA score would predict ROSC with good calibration and discrimination in a cohort of patients from the Helsinki EMS Utstein-style based cardiac arrest registry.

## Methods

### Setting and patient sample

Helsinki, the capital city of Finland has a population of approximately 580,000 inhabitants served by a three-tiered EMS based at eight regional rescue stations. Out-of-hospital resuscitations are attended by a nearest ambulance (BLS, basic life support or ALS, advanced life support) + a physician unit or medical supervisor ALS unit + a first responding rescue unit (i.e., fire engine) when appropriate. Resuscitation is always lead either by the physician or the medical supervisor on-duty. EMS physicians are either specialists or experienced registrars of anaesthesiology with intensive care training. Medical supervisors are specially trained senior paramedics capable of executing ALS procedures and further post-resuscitation care including intubation and ventilatory support, vasoactive medication and sedation, for example. Local resuscitation protocols followed European Resuscitation Council 2005 and 2010 guidelines during the study period [14]. The Helsinki EMS has prospectively collected OHCA data according to the Utstein guidelines since 1994. All data are gathered and validated by Helsinki EMS physicians or on-duty medical supervisors.

In the current retrospective cohort study we included all resuscitation attempts occurring from 1.1.2008 to 31.12.2010. For further analysis we excluded resuscitations where EMS physician decided to stop before resuscitation was commenced or any ALS measures initiated. Refraining from further resuscitation was based on a local do-not-attempt-resuscitation (DNAR) policy, patients medical history (e.g., terminal illness), very long delay on starting CPR, patients of whom CA was due to apparently fatal condition or other rare clinical situation where further resuscitation was considered futile. In the Helsinki EMS cardiac arrest registry, a resuscitation attempt is defined as an attempt of intubation (or other advanced airway), defibrillation or the use of adrenaline and/or amiodarone in addition to chest compressions. Similarly, if ROSC is achieved with chest compressions only resuscitation is considered attempted and the case included in the study.

Notably, Helsinki EMS DNAR guidelines includes situations when resuscitation can be stopped after being initiated. Discontinuing of resuscitation is considered in asystole if the arrest is unwitnessed, if the delay of the ambulance exceeds 10 min from call or if ROSC is not achieved despite 20 min of ALS. Similarly, in witnessed pulseless electrical activity (PEA) resuscitation is stopped if the delay of the ambulance exceeds 15 min, ROSC cannot be achieved within 20 min of ALS or in unwitnessed PEA ROSC is not achieved within 10 min of ALS [15]. These time frames are not applied on hypothermic patients or if the reason for cardiac arrest is drowning or penetrating trauma.

### RACA score

We calculated the RACA score for every patient using previously represented equation: X = 0.3 (constant) + (−0.2× male) + (−0.2× age >80 years) + (−0.6× trauma) + (0.7× hypoxia) + (0.5× intoxication) + (0.6× witnessed by lay people) + (0.5× witnessed by professionals) + (−0.3× nursing home) + (1.2× doctors office) + (0.3× public place) + (0.5× medical institution) + (−0.8× PEA) + (−1.1× asystole) + (0.2× bystander CPR) + (−0.04× minutes until EMS arrival). Probability of ROSC =1/(1+ e^-x^).

In the development of the RACA score the selection of potential predictive factors was performed in a multivariate analysis approach considering available literature. For each categorical variable one condition was defined as standard category, which did not receive a specific coefficient in the model but was defined as a reference for the other conditions of the respected variable. In the original study, following conditions were defined as “standard category”: female gender, age < 80 years, cardiac aetiology, non-witnessed CA, location at home and workplace, ventricular fibrillation as a primary rhythm, and no bystander CPR. Only conditions associated with a negative or positive impact on ROSC were given regression coefficients respectively, and included in the RACA score equation [4].

Notably, there are twenty different possible aetiologies for cardiac arrest in the Helsinki EMS cardiac arrest registry and in RACA equation there are only three (trauma, hypoxia, intoxication). From the twenty possible aetiologies the physician chooses the most probable reason for the cardiac arrest based on clinical findings and patient pre-arrest symptoms on the scene. For this study the following aetiologies found in the Helsinki EMS cardiac arrest registry were included in the RACA category hypoxia: drowning, suffocation, carbon monoxide poisoning, worsening of asthma/chronic obstructive pulmonary disease (COPD), pneumonia and pulmonary embolism. If the presumed aetiology data was missing, the RACA “other” category was used, as in the Helsinki cardiac arrest registry the aetiology of OHCA is presumed to be of cardiac origin unless otherwise stated. Accordingly, the RACA “other” category was also used for all aetiologies other than “trauma”, “hypoxia” or “intoxication”, which are included in the RACA score equation. For example, cardiac arrests with presumable aetiology of non-traumatic bleeding or cerebrovascular disorder were categorised as “other” as those are not included in the RACA score equation. “Trauma” and “intoxication” as aetiologies for OHCA are found as such in the Helsinki EMS cardiac arrest registry. The Helsinki registry summarizes all medical locations and does not include data required for the RACA score, i.e., “nursing home”, “doctor’s office” or “medical institution”. Thus for this study the exact location of these attempts were identified from EMS case notes.

### Primary and secondary outcomes

Primary outcome was of ROSC, defined as palpable pulse at any point during resuscitation for over 30 s.

### Statistical analysis and research permit

Data were analyzed using SPSS version 19.0 (SPSS, Chigaco, Ill., USA). Results are presented as frequencies and percentages or medians (interquartile range). We assessed calibration by comparing observed against predicted ROSC rates by using Hosmer-Lemeshow C test (goodness-of-fit). Expected probabilities of ROSC were calculated using equation of the German study [3] as illustrated above. Discrimination was examined by calculation of the area under receiver operating characteristic (ROC) curve. Area under the curve is presented with 95% confidence intervals. The study plan was approved by the Institutional Review Board of the Helsinki University Hospital. Informed consent was waived due to the retrospective setting of the study.

## Results

After exclusions a total of 680 patients were included in the study and ROSC was achieved in 340 resuscitations (50%). The total incidence of attempted resuscitations was 39/100,000 inhabitants per year. In 377 (36%) cases resuscitation was not attempted and these were excluded from the study.

### Outcome

Baseline characteristics and ROSC rates for different subgroups of patients are presented in Table 1. Primary rhythm was shockable in 275 (40%) of all patients. There were over twice as many male (70%) CA patients as compared to female (30%), but no significant difference on ROSC rates between genders was found (male 48% vs. female 54%, *p* = 0.132). In this study, the age group >80 years was not associated with significantly lower chance of ROSC (age >80 years 48% vs. age <80 years 49%, *p* = 0.91).

**Table 1:** Return of spontaneous circulation for different patient subgroups

	All patients (*N* = 680)	ROSC (*N* = 340)	*P*-value
Demographics			
Age, yrs			
Median	62 (53–73)	62 (55–73)	0.80
< 40	70 (10%)	31 (44%)	0.85
40–49	63 (9%)	30 (48%)	
50–59	128 (19%)	65 (51%)	
60–69	196 (29%)	106 (54%)	
70–79	123 (18%)	59 (48%)	
80–89	81 (12%)	40 (49%)	
90+	19 (3%)	9 (47%)	
Gender			0.132
Female	204 (30%)	111 (54%)	
Male	476 (70%)	229 (48%)	
Initial rhythm			<.001
VF	264 (39%)	175 (66%)	
VT	11 (2%)	9 (82%)	
Asystole	152 (22%)	35 (23%)	
PEA	247 (36%)	115 (47%)	
Unknown	6 (1%)	6 (100%)	
Witnessed			0.045
None	104 (15%)	41 (39%)	
Lay person	421 (62%)	214 (51%)	
Professional	155 (23%)	85 (55%)	
Bystander CPR	341 (50%)	186 (55%)	0.017
	339 (50%)	154 (45%)	
Location			<.001
Home	344 (51%)	147 (43%)	
Ambulance	24 (4%)	15 (63%)	
Workplace	21 (3%)	16 (76%)	
Doctors office	8 (1%)	7 (88%)	
Medical institution	13 (2%)	8 (62%)	
Nursing Home	13 (2%)	5 (38%)	
Public place	257 (38%)	142 (55%)	
Aetiology			<.001
Cardial	405 (60%)	228 (56%)	
Trauma	15 (2%)	8 (53%)	
Hypoxia	55 (8%)	33 (60%)	
Intoxication	32 (5%)	15 (47%)	
Other	173 (25%)	56 (32%)	

Categorical data are shown as counts with percentages in parenthesis and continuous data as medians with 25^th^ and 75^th^ interquartile range points in parenthesis

*VF* ventricular fibrillation, *VT* ventricular tachycardia, *PEA* pulseless electrical activity, *CPR* cardiopulmonary resuscitation

### RACA score

**Fig. 1:**
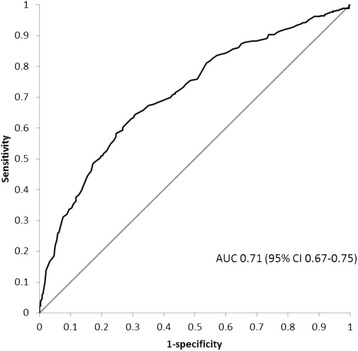
Receiver operating characteristic curve for prediction of ROSC. AUC: area under the curve, CI: confidence interval

**Fig. 2:**
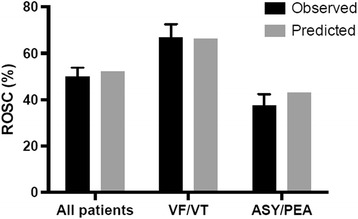
Observed and predicted ROSC rates for all patients and divided by initial rhythm with 95% confidence interval

**Fig. 3:**
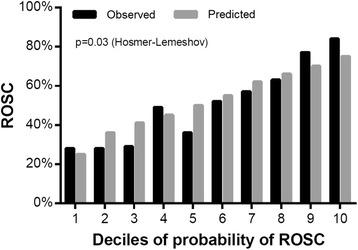
Concordance between observed and predicted ROSC rates for all patients divided into deciles

The RACA score was higher in patients with ROSC (0.62 [0.46–0.69] than in those without (0.46 [0.36–0.57]) (*p* < 0.001). For the entire study population RACA score showed moderate discrimination (AUC 0.71, CI 0.67–0.75) for predicting ROSC (Fig. 1). Predicted ROSC rate in the whole sample was 52% while observed ROSC rate was 50% (CI 46–54). The mean predicted ROSC rate for the 275 resuscitations with shockable initial rhythm was 66% and ROSC was achieved in 67% (CI 61–73) of the patients. For patients with a non-shockable rhythm results were 43% vs. 38% (CI 33–42), respectively (Fig. 2). Observed and predicted ROSC with the RACA indicated reasonable calibration overall (*p* = 0.30), with better calibration in patients with a shockable initial rhythm (*p* = 0.75) than with a non-shockable rhythm (*p* = 0.04). Concordance between observed and predicted ROSC rates divided into deciles are shown in Fig. 3. In the whole study population RACA score tended to overestimate ROSC rates in general while observed ROSC rates were markedly higher in the two highest deciles. Similarly, the score underestimated ROSC rates in the highest deciles in both shockable and non-shockable rhythms but for non-shockable rhythms outcome tended to be worse than predicted.

No difference between observed and predicted ROSC rates in resuscitations attended by a specialist (50% vs 53%, CI 45–55) or registrar (55% vs 53%, CI 48–62) was found, but rates were lower than predicted in resuscitations lead by a medical supervisor (36% vs 49%, CI 25–47). The use of adrenalin was associated with lower than predicted ROSC rate (41% vs 50%, CI 37–45). We found no influence whether ALS unit delay was over (44% vs 48%, CI 39–50) or under (54% vs 55%, CI 49–59) median time or if rescue unit was the first responder (49% vs 51%, CI 38–61) or not (50% vs 52%, CI 46–54). Observed and predicted ROSC rates for subgroups of patients are shown in Table 2.

**Table 2:** Observed and predicted ROSC rates for different patient subgroups

Factor	Patients (n)	Observed ROSC (95% CI;%)	Predicted ROSC (%)	Impact
Team leader				
Specialist	392	50.3 (45.4–55.3)	52.6	Neutral
Registrar	210	54.8 (48.1–61.5)	52.7	Neutral
Medical supervisor	78	35.9 (25.2–46.6)	49.1	Negative
Primary rhythm				
VF/VT	275	66.9 (61.3–72.5)	65.8	Neutral
PEA/Asystole	399	37.6 (32.8–42.4)	43.4	Negative
Rescue unit as first responder				
Yes	69	49.3 (37.5–61.1)	50.9	Neutral
No	611	50.1 (46.1–54.1)	52.4	Neutral
Delay to ALS unit				
< median	389	54.2 (49.2–59.1)	55.1	Neutral
> median	291	44.3 (38.6–50.0)	48.4	Neutral
Adrenaline				
Yes	513	41.1 (36.8–45.3)	50.2	Negative
No	167	77.2 (70.8–83.6)	58.5	Positive

*VF* ventricular fibrillation, *VT* ventricular tachycardia, *PEA* pulseless electrical activity, *CPR* cardiopulmonary resuscitation, *ALS* advanced life support, *ROSC* return of spontaneous circulation

## Discussion

In this study, we validated RACA score externally in a physician staffed urban EMS system and found good overall calibration and moderate discrimination. We did, however, find that the score had suboptimal calibration in patients with non-shockable primary rhythm. To the best of our knowledge, this is the first study externally that validates the RACA score in an EMS system.

Developing a practical severity-of-illness scoring system for out-of-hospital cardiac arrest patients would allow patient heterogeneity adjustment and measurement of quality of care in analogy to commonly used severity of illness scoring systems such as APACHE (Acute Physiology and Chronic Health Evaluation) and SAPS (Simplified Acute Physiology Score) developed for the similar purposes for the general intensive care unit population [16, 17]. This would allow comparisons between different EMS systems and patient cohorts as baseline factors affecting outcome would be taken into account. However, scoring system updates are also often required, as performance of the score tends to deteriorate over time due to changes in casemix and patient management. This is common practise in the use of severity of illness scores in intensive care, where the commonly used APACHE IV and SAPS III scores have reached their third and fourth generation, respectively [18]. The RACA score was developed in 2011 based on prospectively registered data from patients treated between 1998 and 2008, and may indeed benefit from a recalibration in the future or with more recent data. For example, failure of prehospital ROSC with ongoing CPR on admission is considered as a negative outcome [4]. Developments of OHCA management include extra-corporeal membrane oxygenation (E-CPR) and out- and in-hospital transfers with ongoing (mechanical) CPR to the cardiac catheterization laboratory for intra-arrest interventions [19]. It is highly likely that such changes will influence the accuracy of the RACA score.

The purpose of the RACA score was to derive a score enabling prediction of initial resuscitation success adjusted to different conditions, using data available on EMS arrival at the scene [4]. External validation of such score in other population is necessary before prediction model can be used more widely, as the model reflects the risk of a patient in the system and population in which it was developed. The original study by Gräsner et al. was based on data from German Resuscitation Registry and its applicability has only been tested in a German populations and EMS systems [4]. However, transferring RACA score to another country with different population and EMS system might affect the performance and generalizability of the score.

Compared with the original study by Gräsner et al. [4] our cohort was different in terms of primary rhythm (ventricular fibrillation (VF) 28% vs 39%, pulseless electrical activity (PEA) 11% vs 36%, asystole (ASY) 46% vs 22%), non-witnessed CA (41% vs 15%), bystander cardiopulmonary resuscitation (CPR) (15% vs 50%) and ROSC rate (44% vs 50%). Nonetheless, we found a similar performance of the RACA score in terms of discrimination (AUC 0.73 vs. 0.71) supporting the generalizability of the score. Overall, observed ROSC rates corresponded well with predicted rates from the RACA score and especially well within the shockable rhythm -subgroup. In patients with a non-shockable primary rhythm the score showed poor calibration. One reason for this could be the do-not-attempt resuscitation (DNAR) practice of Helsinki EMS, limiting of both the number of resuscitation attempts included in this study and the duration of resuscitation attempts -especially in non-shockable rhythms [15]. This most likely reduces the ROSC rate in our population as in the original study by Gräsner et al. there was a ROSC rate of approximately 35% in patients with EMS delay of 20 min or more from the onset of CA –situation where resuscitation would be stopped for non-shockable rhythms according to Helsinki EMS DNAR practice [4]. A previously published study showed unpredicted survivors especially among patients with unwitnessed asystole when the Helsinki EMS DNAR practice was followed -although, the prognosis in this subgroup seems very poor in general [15]. This might to some extent explain poor calibration found in this subgroup.

The RACA score has been used in German studies since its development. In a previously published study RACA score was used to compare predicted and observed non-traumatic out-of-hospital cardiac arrest ROSC rates over three 5-year time periods in the EMS of Bonn, Germany, showing lower than observed ROSC rates in all time periods [20]. It was also used in another German study to compare ROSC rates in out-of-hospital cardiac arrest patients with difficult or unsuccessful intubations where predicted and observed ROSC rates were similar in the group of difficult intubations, while the unsuccessful intubation group had lower than predicted ROSC rates [21]. ROSC rate was higher than predicted by the RACA score in a study comparing seven different centres in Germany when used as one part of EMS quality assessment [22]. In general, RACA score has showed slight tendency to underestimate rather than overestimate ROSC rates in German cohorts, whereas in our study it rather tended to overestimate ROSC rates –except in patients with high probability of ROSC.

We found no influence between observed and predicted ROSC rates in resuscitations lead by specialist or registrar as was the case in the original study between different specialties of the physician. Similarly, there was no influence whether first responder was rescue unit or not, or if delay of the ALS unit was over vs. under median time. However, we found a negative impact on resuscitations lead by a medical supervisor. This might partly be due to the fact there being two persons less on scene as opposed to resuscitations attended by a physician staffed unit. Furthermore, in comparison with EMS physicians, medical supervisors had more limited exposure and experience regarding ALS procedures and cardiac arrest patients in overall. During the study period, medical supervisors attended to 78 OHCA patients compared to 602 EMS physician lead resuscitations, as shown in Table 2. There was also a negative impact on adrenaline use during resuscitation, which could be explained by the lower than expected ROSC rates in patients with non-shockable rhythms. Similarly, the positive impact on not using adrenaline is most likely explained by the high prevalence of shockable rhythm in our cohort as ROSC is often achieved by defibrillation only -supported by high ROSC rate of 77% in this subgroup.

There are some limitations of our study. Firstly, as a principle, all OHCAs in the Helsinki cardiac arrest registry are presumed as of cardiac origin unless otherwise stated, and in this study “other” category was used accordingly, as only “intoxication”, “trauma” and “hypoxia” have significant impact on the probability of ROSC in RACA score equation. Some selection bias due to incorrect presumed aetiology cannot, however, be ruled out.

Secondly, the duration of each resuscitation was not observed although it may have impact on the probability of ROSC. This may be highlighted when comparing EMS systems using different protocols in regards to cessation of a resuscitation attempt. However, this variable is not included in the RACA score equation and was therefore out of scope in this study.

## Conclusions

This study conducted in one urban EMS system found a good overall calibration and moderate discrimination of the RACA score suggesting external validity of the score. Calibration was better in patients with a shockable rhythm and suboptimal in patients with non-shockable rhythm which can be due to a local do-not-attempt-resuscitation policy. The lower than expected overall ROSC rate in resuscitations attended by medical supervisors requires further study. Suboptimal calibration in certain subgroups of patients and only moderate discriminative power may limit the use of RACA score in different EMS systems.

## Abbreviations

ALS: Advanced life support
APACHE II: Acute physiology and chronic health evaluation II
ASY: Asystole
BLS: Basic life support
CPR: Cardiopulmonary resuscitation
DNAR: Do not attempt resuscitation
EMS: Emergency medical service
OHCA: Out-of-hospital cardiac arrest
PEA: Pulseless electrical activity
RACA: Return of spontaneous circulation after cardiac arrest
ROSC: Return of spontaneous circulation
SAPS II: Simplified acute physiology score ii
VF: Ventricular fibrillation
VT: Ventricular tachycardia
